# Prognostic Performance of Three Lymph-Node Staging Systems on Gastric Signet-Ring-Cell Carcinoma

**DOI:** 10.3390/cancers15123170

**Published:** 2023-06-13

**Authors:** Limin Zhang, Yan Ma, Bao Liu

**Affiliations:** 1 Department of Gastroenterological Surgery, Harbin Medical University Cancer Hospital, Harbin Medical University, Harbin 150081, China; 831226@hrbmu.edu.cn (L.Z.); doctormayan@163.com (Y.M.); 2 The First Department of Medical Oncology, Harbin Medical University Cancer Hospital, Harbin 150081, China

**Keywords:** gastric signet cell carcinoma, lymph-node metastases, LODDS, prognosis

## Abstract

**Simple Summary:**

Signet-ring-cell carcinoma (SRCC) is a specific subtype of gastric cancer with a lower incidence and poor prognosis. At the same time, the prognosis of early SRCC and late SRCC remains controversial. Early SRCC and SRCC have different prognostic features, especially lymph-node metastasis. Therefore, the accurate assessment of lymph-node metastasis is important for SRCC. We retrospectively analyzed the prognostic value of different lymph-node staging systems for early and advanced SRCCs. We demonstrated that the predictive performance of log odds of positive lymph nodes (LODDS) is superior to pN-stage and lymph-node metastasis rate (LNR) regardless of early or late SRCC, and is an independent risk factor associated with patient outcomes. This provides a theoretical basis for the further exploration of lymph-node metastasis in SRCC.

**Abstract:**

Background: The lymph-node staging system can predict the prognosis of gastric signet-ring-cell carcinoma (SRCC). However, there are significant differences in lymph-node status between early SRCC and advanced SRCC. Additionally, the optimal system for early and advanced SRCC remains unknown. Methods: This study retrospectively analyzed 693 SRCC patients who underwent radical resection in the Department of Gastrointestinal Surgery, Harbin Medical University Cancer Hospital. The predicted performance of three lymph-node staging systems, including pN staging, lymph-node metastasis rate (LNR), and log odds of positive lymph nodes (LODDS), was compared using the receiver characteristic operating curve (ROC) and c-index. The Kaplan–Meier method and the log-rank test analyzed the overall survival of patients. The Cox risk regression model identified independent risk factors associated with patient outcomes. The nomogram was made by R studio. Results: The 693 SRCC included 165 early SRCC and 528 advanced SRCC. ROC showed that LODDS had better predictive performance than pN and LNR in predicting prognosis regardless of early or advanced SRCC. LODDS can be used to predict the prognosis of early and advanced SRCC and was an independent risk factor associated with patient outcomes (*p* = 0.002, *p* < 0.001). Furthermore, the nomogram constructed by LODDS and clinicopathological features had good predictive performance. Conclusions: LODDS showed clear prognostic superiority over both pN and LNR in early and advanced SRCC.

## 1. Introduction

Gastric cancer (GC) has the fifth highest incidence and the is third leading malignancy in cancer death, causing approximately 780,000 deaths annually [1]. Although the incidence of GC is decreasing, the incidence of signet-ring-cell carcinoma (SRCC) is increasing [2], accounting for 35% to 45% of adenocarcinomas in Asia, Europe and the United States [3]. The World Health Organization describes gastric ring-cell carcinoma (SRCC) based on tumor microtissue characteristics [4]. SRCC is defined as a cytoplasmic abundant and mucus-filled tumor cell under pathological detection, with the nucleus squeezed on one side of the cytoplasm to have a string-like appearance, and the main component (more than 50% of the tumor) consists of isolated or small groups of malignant cells containing intracytoplasmic mucus [4,5]. It is worth noting that compared with non-SRCC, the biological behavior of SRCC is significantly heterogeneous due to the tumor infiltration depth. Gastric Cancer showed that early SRCCs have a better prognosis than non-SRCCs, while advanced SRCCs have a lower prognosis than non-SRCCs, and early SRCCs and advanced SRCCs have different prognoses and clinical features [5]. Furthermore, a meta-analysis suggested that the frequency of lymph-node metastass in early SRCC is lower than that non-SRCC, while there was no significant difference in the frequency of lymph-node metastasis between advanced SRCC and non-SRCC [6]. This suggests that early SRCC may have different disease processes than advanced SRCC. Therefore, given the complex lymphatic drainage anatomy around the stomach, an in-depth analysis of the lymphatic status of SRCC is helpful in furthering the understanding of the disease process, and there is still a need to find more reliable, specific clinical models to predict clinical outcomes for SRCC.

Lymph-node metastasis is one of the most important prognostic indicators for GC [7]. At present, the most widely used lymph-node evaluation method in clinical practice is the pN stage based on the number of metastatic lymph nodes (mLNs), developed by the American Joint Committee on Cancer (AJCC), which is also the basis for the pTNM stage [8]. However, the N staging is affected by the number of lymph nodes removed (RLNs), which can cause stage migration if RLNs are insufficient [9]. Hence, in order to accurately predict prognosis, modified nodal staging systems, such as lymph-node metastasis rates (LNRs) based on mLNs/RLNs, and log odds of positive lymph nodes (LODDS) can theoretically be used as an alternative to pN staging due to the prognostic effects of both mLNs and RLNs [9]. In addition, large survivorship data based on the surveillance, epidemiology, and final outcome (SEER) database provide evidence for the clinical application of LNR and LOODS, and the results suggest that different lymph-node staging systems can predict the prognosis of GC patients well [10]. However, there are still some differences in the predictive performance and applicability of different nodal staging systems [10,11,12], and there are few studies on the nodal staging system of SRCC. Therefore, considering the frequency of lymph-node metastasis between early SRCC and advanced SRCC, in addition to exploring the effectiveness of different nodal staging systems in predicting SRCC prognosis, selecting an effective lymph-node staging system based on the biological behavior of SRCC will help to accurately predict patient prognosis.

In this study, we compared the prognostic performance of the pN, LNR, and LODDS nodal staging system for early SRCC and advanced SRCC based on the lymph-node status of early SRCC and advanced SRCC, respectively, to determine the optimal nodal staging system for predicting overall survival in patients.

## 2. Materials and Methods

### 2.1. Patients

This study retrospectively analyzed patients who underwent R0 resection and D2/D2 + lymph dissection in the Department of Gastrointestinal Surgery of Harbin Medical University Cancer Hospital from January 2014 to January 2017, and were pathologically diagnosed with SRCC. SRCC was diagnosed by two experienced pathologists based on pathological tissue. SRCC was defined as the main component (more than 50% of the tumor) consists of isolated or small groups of malignant cells containing intracytoplasmic mucus in pathological tissues. Exclusion criteria: (1) preoperative chemotherapy; (2) preoperative radiotherapy; (3) other systemic malignant tumors; and (4) severe infection. In reference to the classification in the previous study, early gastric cancer is defined as a gastric tumor that has invaded no more deeply than the submucosa layer (T1), irrespective of the lymph-node metastasis status. Advanced gastric cancer is defined as a tumor that has invaded at least to the muscle layer (T2–T4) [5]. Surgery and lymph-node dissection followed the fifth edition of Japanese guidelines for the treatment of gastric cancer [13]. Informed consent was obtained from all patients before surgery. The study was approved by the Ethics Committee of Harbin Medical University Cancer Hospital.

The patient’s clinical pathological data were stored in the case management system of the Affiliated Cancer Hospital of Harbin Medical University, including the basic characteristics of the patient, pathological examination, etc. Patients received regular follow-up visits by phone, WeChat, email, or at Harbin Medical University Cancer Hospital after discharge. Stage I patients were followed every 12 months, stage II patients were followed up every 6 months, and stage III patients were followed every 3–6 months.

### 2.2. Lymph-Node Staging System

The pN stage was determined by the number of positive lymph nodes under pathologic diagnosis and was consistent with the eighth edition of AJCC staging [8]. pN0 was 0 mLNs, pN1 was 1–2 mLNs, pN2 was 3–6 mLNs, and pN3 was ≥7 mLNs. LNR was the ratio of the number of positive lymph nodes to the number of removed lymph nodes, ranging from 0 to 1. Given the different frequencies of lymph-node metastasis between early SRCC and advanced SRCC, we used different classifications for LNRs. We found that 78.8% of early SRCC did not have lymph-node metastasis, and the frequency of early SRCC lymph-node metastasis was low. After referring to the LNR cutoff point of the previous studies [10,14], finally, we adopted 0.1 as the cut-off point, LNR of 0.0–0.1, >0.1. For advanced SRCC, due to the high frequency of lymph-node metastasis, we used 0.2 as the cut-off point, LNR of 0–0.2, 0.2–0.4, 0.4–0.6, and >0.6. LODDS was calculated as log([pLN + 0.5]/[nLN + 0.5]), where pLN is the number of positive lymph nodes, nLN is the number of negative lymph nodes, and the numerator and denominator were added by 0.5 to avoid singularity. nLN was calculated as the number of removed lymph nodes minus the number of positive lymph nodes. For the classification of LODDS, we referred to the cut-off points of previous studies [10], which were classified as LODDS 0 ≤ −1.5, −1.5 < LODDS 1 ≤ −1.0, −1.0 < LODDS 2 ≤ −0.5, −0.5 < LODDS 3 ≤ 0, LODDS 4 > 0.

### 2.3. Statistical Analysis

Overall survival (OS) was defined as the time to follow-up from the time of surgery, to time of death or last survival, expressed by 5-year survival rate. Overall survival was estimated using the Kaplan–Meier method and differences in survival were assessed using the log-rank test. The chi-square test was used to analyze correlations between clinicopathological features. A receiver operating characteristics (ROC) curve was used to analyze the area under the curve (AUC), and to compare the accuracy of different nodal staging systems. In addition, we used the c-index to compare the predictive performance of different lymph-node systems: the higher the c-index, the better the prediction performance. The Spearman coefficient and the two-tailed test were used to assess the relevance of the nodal staging system. Risk ratios (HRs) and 95% confidence intervals (95% CI) were calculated using the Cox proportional hazards model, and statistically significant parameters in the univariate analysis were incorporated into a multivariate analysis to determine independent risk factors related to patient prognosis. The nomogram models were drawn through the R studio by ‘SvyNom’ and ‘rms’ packages. SPSS Windows 25.0 was used for statistical analysis, and *p* < 0.05 was considered statistically significant.

## 3. Results

### 3.1. Clinical Characteristics

Finally, this study included 165 patients with early SRCC and 528 patients with advanced SRCC (Table 1). The median age of early SRCC and advanced SRCC were 55 and 59. For tumor aggressiveness, compared with early SRCC, advanced SRCC had a larger tumor diameter (*p* < 0.001), higher proportion of vascular invasion (*p* < 0.001) and nerve invasion (*p* < 0.001), and the frequency of lymph-node metastasis (*p* < 0.001) was significantly higher than that of early SRCC.

### 3.2. Characteristics of Different Lymph-Node staging Systems

**Figure 1:**
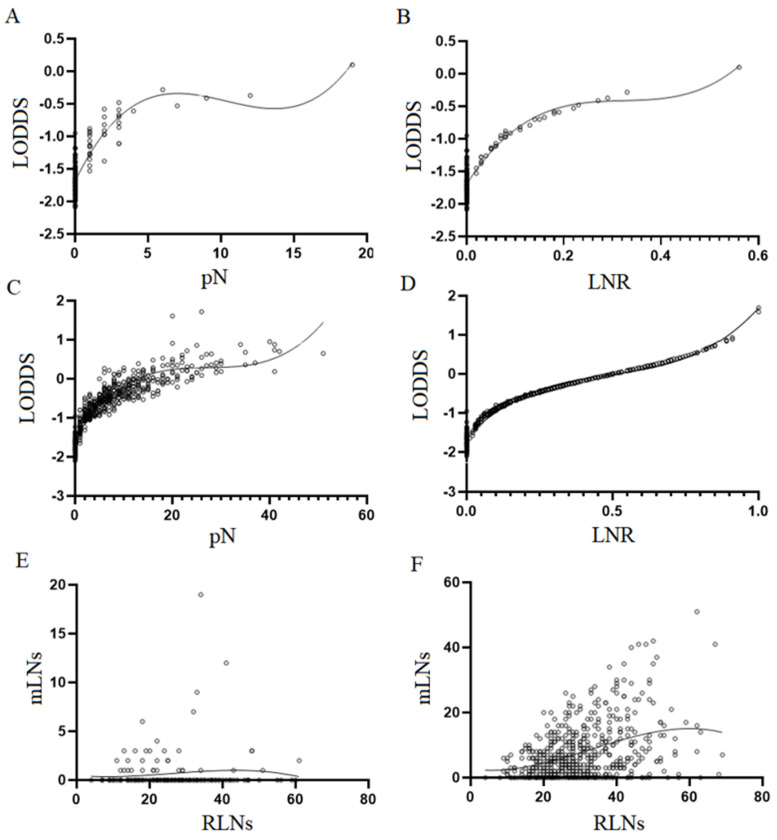
Scatterplot and linear relationship of lymph-node staging system. (**A**) scatter plot of the relationship between LODDS and pN for T1 SRCC. (**B**) Scatter plot of the relationship between LODDS and LNR for T1 SRCC. (**C**) Scatter plot of the relationship between LODDS and pN for T2–T4 SRCC. (**D**) Scatter plot of the relationship between LODDS and LNR for T2–T4 SRCC. (**E**) Scatter plots of the relationship between RLNs and mLNs for T1 SRCC. (**F**) Scatter plot of the relationship between RLNs and mLNs for T2–T4 SRCC.

We analyzed scatterplots of LOODS versus two other nodal staging systems. For early SRCC, LODDS was more highly correlated with LNR (*p* < 0.001) than pN (*p* < 0.001) (*r* = 0.694 vs. 0.690, *r*: spearman coefficient) (Figure 1A,B). For advanced SRCC, LODDS was more highly correlated with LNR (*p* < 0.001) than pN (*p* < 0.001) (*r* = 0.997 vs. *r* = 0.942, *r*: spearman coefficient) (Figure 1C,D). Obviously, regardless of early or advanced SRCC, the values of LODDS are scattered when no lymph-node metastasis occurs (pN/LNR = 0). Similarly, the values of LODDS are still scattered when LNR = 1.

In addition, we analyzed the scatterplots of RLNs and mLNs, and the results showed that the correlation between RLNs and mLNs in advanced SRCC was higher than that of early SRCC (*r* = −0.040 vs. *r* = 0.342, *p* = 0.607, *p* < 0.001, *r*: spearman coefficient) (Figure 1E,F). This suggests that staged migration is more likely to occur in advanced SRCC than in early SRCC.

### 3.3. Prognostic Performance of Different Node Staging Systems

**Figure 2:**
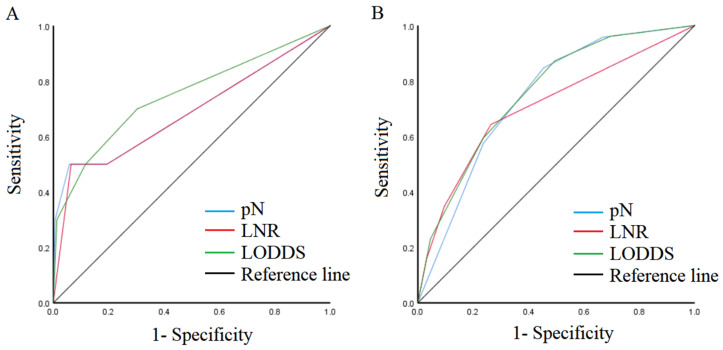
Comparison of ROC of the nodal staging system. (**A**) T1 SRCC. (**B**) T2–T4 SRCC.

To compare the predictive performance of the nodal staging systems, ROC showed that the AUCs of pN, LNR, and LODDS for early SRCC were 0.694 (95% CI: 0.485–0.904), 0.685 (95% CI: 0.480–0.891), and 0.748 (95% CI: 0.563–0.933) (Figure 2A). For advanced SRCC, the AUCs of pN, LNR, and LODDS were 0.741 (95% CI: 0.697–0.785), 0.707 (95% CI: 0.663–0.751), and 0.756 (95% CI: 0.714–0.798) (Figure 2B).

**Figure 3:**
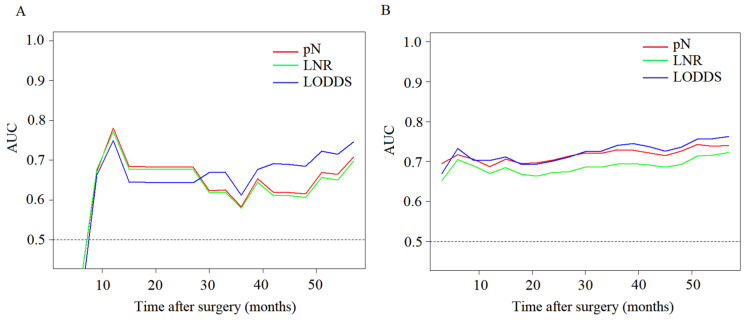
Comparison of T-ROC of the nodal staging system. (**A**) T1 SRCC. (**B**) T2–T4 SRCC.

We also performed a T-ROC analysis and the results showed AUCs of pN, LNR, and LODDS were 0.781, 0.772, and 0.749 for one year postoperative for early SRCC, and the AUCs of pN, LNR, and LODDS were 0.583, 0.579, and 0.612 for three years postoperative for early SRCC (Figure 3A). For advanced SRCC, the AUCs of pN, LNR, and LODDS were 0.687, 0.670 and 0.703 for one year postoperative, and the AUCs of pN, LNR, and LODDS were 0.729, 0.694 and 0.741 for three years postoperative (Figure 3B). Additionally, the results show that the predictive performance of LODDS gradually improves over time.

Considering the potential impact of the number of lymph nodes removed on the number of lymph node metastases, we assessed the predictive performance of the three nodal staging systems based on different numbers of lymph nodes retrieved. The results showed that LODDS had the highest c-index. This indicated that LODDS performed better than pN and LNR in both early and late SRCC, and regardless of the number of lymph nodes retrieved (Table 2).

### 3.4. The Effect of LODDS on Patient Survival

**Figure 4:**
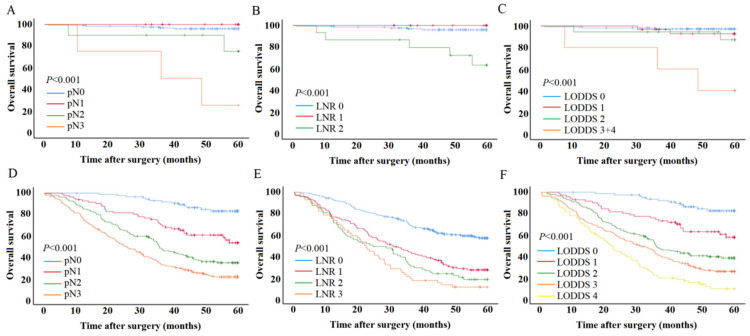
Survival curves of the lymph-nodal staging system. (**A**–**C**) T1 SRCC. (**D**–**F**) T2–T4 SRCC.

After finding that LODDS has the highest predictive performance, we used LODDS to predict the prognosis of early and advanced SRCC. For early SRCC, the 5-year survival rates for pN0–pN3 were 96%, 100%, 75%, and 25% (*p* < 0.001). The 5-year survival rates for LNR 0–2 were 96.0%, 100%, and 63.2% (*p* < 0.001). The 5-year survival rates for LODDS 0-LODDS 3 + 4 were 97.2%, 92.7%, 87.2%, and 40.0% (*p* < 0.001) (Figure 4A–C). For advanced SRCC, the 5-year survival rates for pN0–pN3 were 82.9%, 52.9%, 34.0%, and 20.6% (*p* < 0.001). The 5-year survival rates for LNR 0–4 were 56.6%, 26.5%, 17.5%, and 10.3% (*p* < 0.001). The 5-year survival rates for LODDS 0–4 were 82.5%, 57.4%, 37.8%, 25.1%, and 8.7% (*p* < 0.001) (Figure 4D–F).

### 3.5. Univariate and Multivariate Analysis for Patient Outcomes

Considering that pN, LNR, LODDS calculation formulae contain the same parameters, we performed Cox analysis on pN, LNR, and LODDS, respectively. The results showed that for early SRCC, pN, LNR, and LODDS were all identified as independent prognostic factors related to patient prognosis (Table 3). For advanced SRCC, pN, LNR, and LODDS were identified as independent risk factors related to patient outcomes (Table 4).

### 3.6. Nomogram in the Patient Outcomes

**Figure 5:**
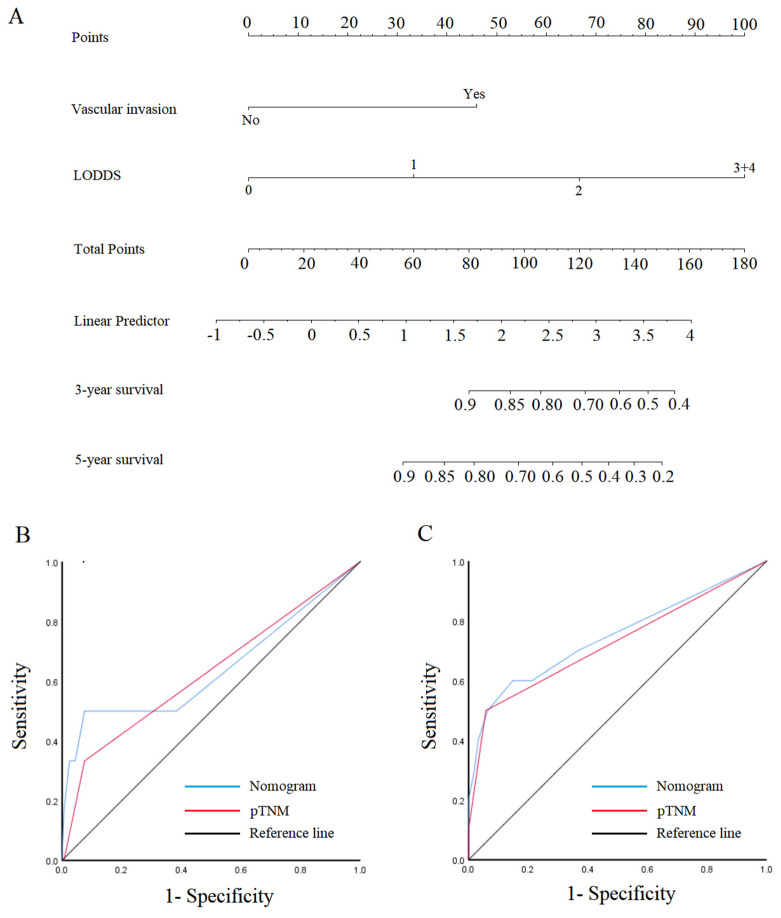
Nomogram and ROC curves to predict patient prognosis. (**A**) Nomogram for T1-SRCC. (**B**,**C**) ROC analyzes the ability of nomogram to predict 3- and 5-year prognosis in T1 SRCC.

**Figure 6:**
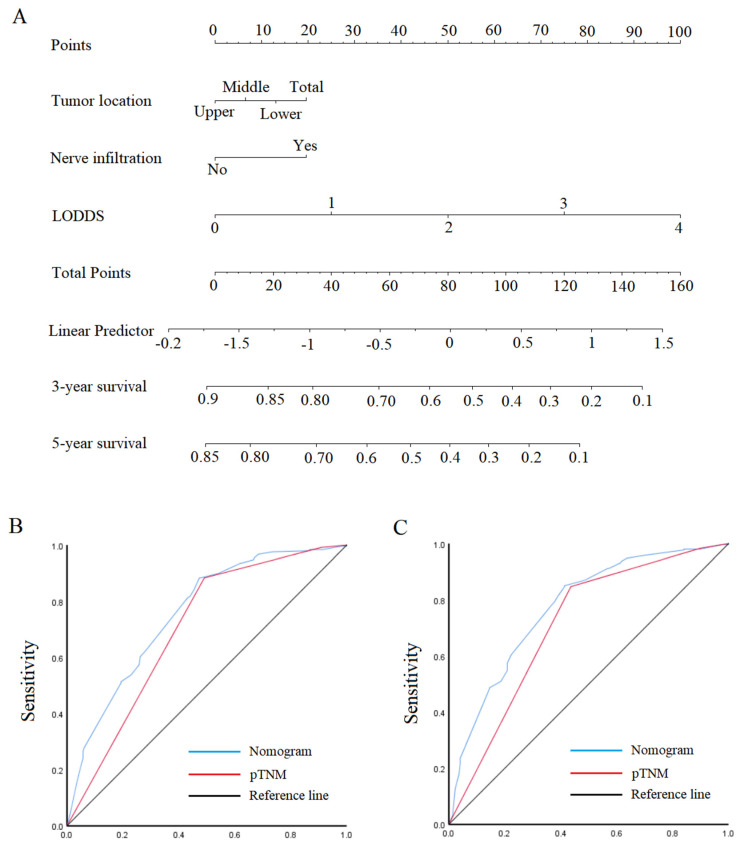
Nomogram and ROC curves to predict patient prognosis. (**A**) Nomogram for T2–T4 SRCC. (**B**,**C**) ROC analyzes the ability of nomogram to predict 3- and 5-year prognosis in T2–T4 SRCC.

Since pN, LNR, and LODDS were independent risk factors associated with the prognosis of patients with early SRCC and advanced SRCC. Considering the difference in predicted performance of pN, LNR, and LODDS, we finally selected LODDS with the best predictive performance to construct nomograms (Figure 5A and Figure 6A). For early SRCC, the AUCs of nomogram were 0.641 (95% CI: 0.354–0.928) and 0.747 (95% CI: 0.552–0.941) for 3- and 5-year prognosis, the sensitivity was 50% and 60%, and the specificity was 92.5% and 85.2% (Figure 5B,C). For advanced SRCC, the AUCs of nomogram were 0.756 (95% CI: 0.715–0.796) and 0.773 (95% CI: 0.732–0.813) for 3- and 5-year prognosis, the sensitivity was 88.4% and 85.1%, and the specificity was 52.7% and 58.6% (Figure 6B,C).

## 4. Discussion

Accurate staging systems are essential for predicting long-term survival of cancer patients. Due to the importance of LNs status in prognosis after GC resection, there is still considerable interest in defining the optimal LNs stage. Based on differences in lymph-node status between early and advanced SRCC [6], we compared different nodal staging systems and found that LODDS had better predictive performance than pN and LNR in early and advanced SRCC.

The risk of lymph-node metastasis is low when SRCC is confined to the mucosal layer, and significantly increased when SRCC penetrates the submucosa to the deeper layers [15]. This may explain the finding of Kao et al. that the frequency of lymph-node metastasis in early SRCC is not significantly different from that of non-SRCC, but the frequency of lymph-node metastasis in advanced SRCC is higher than that of non-SRCC [5]. However, meta-analyses suggested that the frequency of lymph-node metastasis in early SRCC was lower than that in non-SRCC, whereas there is no significant difference in the frequency of lymph-node metastasis between advanced SRCC and non-SRCC [6], and some heterogeneity in studies is inevitable despite efforts to ensure homogeneity in the included studies. These heterogeneities may have contributed to conflicting views of SRCC lymph-node metastasis. Therefore, our current research focuses on selecting an appropriate evaluation tool for early and advanced SRCC lymph-node metastasis with known clinical information, rather than exploring the root cause of heterogeneity. Importantly, we found that the predictive performance of pN, LNR, and LODDS increases over time, both early and advanced SRCC, which is also consistent with previous studies [16]. Obviously, the staging system of lymph nodes is important for the long-term prognosis of patients. Choosing the appropriate evaluation tool can also help to individualize and more accurately predict the prognosis of SRCC patients.

Theoretically, LODDS may be a superior staging scheme because LODDS has more information than pN and has greater resolving power than LNR. pN represents the absolute number of mLNs, and LNR represents the combined information of mLNs and RLNs. It appears that LNR versus LODDS is more reasonable than pN staging because mLNs are highly dependent on RLNs, whereas the optimal extent of lymph node resection and the mean number of RLNs in GC resection vary widely. There are still significant differences in the degree of anatomy and analysis of LNs in patients in East–West surgical centers [12]. Importantly, insufficient RLNs can lead to stage migration, and LNR and LODDS are better options to avoid phased migration. However, due to certain limitations, LNR cannot be considered an alternative to pN staging, first, there is no difference in survival between pN and node-negative patients in the LNR system. Second, there are differences in the classification of LNRs in different studies [17,18]. In this study, 78.8% of patients with early SRCC did not have lymph-node metastasis, and considering that early SRCC rarely occurs lymph-node metastasis, the same LNR classification method as advanced SRCC may not increase LNR discrimination. Therefore, we adjust the cut-off points of LNRs of early SRCC according to the classification of previous LNRs. However, the predictive performance of LNR is still lower than LODDS. Finally, for some patients with non-negative lymph nodes (pN0/LNR ≠ 0), the higher the RLNs, the higher the true negative rate of mLNs, thereby reducing the risk of death. Similarly, for patients whose retrieved nodes were all positive (LNR = 1), increasing RLNs meant a further increase in the probability of positive lymph nodes, predicting a worse prognosis. As a result, LODDS utilizes all available information that pN and LNR do not.

In addition, we evaluated the predictive performance of different nodal staging systems based on different number of lymph nodes retrieved. First, at least 16 lymph nodes need to be retrieved under the eighth edition of staging to meet adequate nodal staging. However, there are still some patients who have fewer than 16 lymph nodes retrieved, this also means that patients at this stage may undergo staged migration. We found that LODDS still outperformed LNR and pN in patients with an insufficient number of removals, suggesting that LODDS has good applicability for patients who may have stage migration. Second, when the lymph nodes are sufficiently retrieved, LODDS still has the best predictive performance. In addition, we found through T-ROC that the predictive performance of LODDS gradually improved over time. These results indicate that LODDS has good clinical value and is worthy of further application.

Patients without lymph node metastases are clinically classified as pN0, which also leads to the underlying hypothesis that patients with the same pN may have the same prognosis regardless of the number of RLNs. The fact that large studies have shown that the risk of death in pN0 patients is not constant means that pN classification may not accurately predict clinical outcomes in large patients [11]. Therefore, the ability of pN and LNR to be used in node-negative SRCC will be greatly limited. LODDS is calculated using empirical transformations that prevent singularity caused by zero observation and are the smallest deviation estimates of true logarithmic probabilities [19]. Given these statistical characteristics, LODDS can better distinguish heterogeneity in patients without lymph-node metastasis (pN0, LNR = 0) or LNR = 1. It is important to note that the correlation is not linear, and for early SRCC, LODDS increases more slowly and stabilized when LNR is between 0.2–0.4. In contrast, when the LNR is less than 0.2, a steeper curve can be observed, which further confirms the heterogeneous process of lymph-node metastasis. This suggests that LODDS has greater discriminating power in patients with very low LNR. In particular, patients with LNR = 0 still have heterogeneous LODDS even with the same prognosis. Therefore, LODDS has good discrimination for early SRCC and is a reliable prognostic stratification tool.

For advanced SRCC, we also observed nonlinear relationships, suggesting that survival heterogeneity of the same pN stage or the same LNR still exists in advanced SRCC. As with early SRCC, LODDS provides good discrimination in patients who have not developed lymph node metastases. In particular, the phenomenon of LNR = 1 in advanced SRCC also greatly limits the use of LNR. Importantly, because advanced SRCC are more prone to lymph-node metastasis, insufficient RLNs or insufficient examination of lymph nodes can lead to stage migration [20,21]. In addition, we found that the tumor size, vasculature invasion, and proportion of neural infiltration in advanced SRCC were significantly higher than those in early SRCC, and these factors are also important tumor features affecting SRCC lymph-node metastasis [22,23]. Additionally, we found that the correlation between RLNs and mLNs in advanced SRCC is higher than that of earlier SRCC, so we speculate that advanced SRCC may be more prone to staged migration. Clearly, LODDS has the advantage of advanced SRCC in that it can identify heterogeneous populations with LNR = 1 and avoid the effect of prediction bias due to stage migration [24]. Furthermore, we also found that pN, LNR, and LODDS were independent risk factors related to patient prognosis, which fully illustrates the important impact of lymph-node metastasis on patient prognosis. This also indirectly reflects the prognostic importance of RLNs, which are also valuable for patient outcomes [25,26]. In summary, whether early or advanced SRCC, adequate RLNs are an important means to ensure accurate staging and improve patient outcomes.

In clinical work, pTNM staging based on tumor anatomy provides clinicians with useful but incomplete prognostic information. Even at the same stage, there are still some differences in the prognosis of patients. Line-plots based on multifactorial analysis and integrating multiple clinical indicators help quantify the prognostic risk of patients and further provide detailed risk stratification. Li et al. constructed a nomogram based on lymph-node status and age to predict the prognosis of patients with GC [27]. Xu et al. constructed a nomogram based on LODDS and clinicopathological features of patients to predict the prognosis of SRCC patients [16]. Therefore, considering the difference in lymph-node metastasis between early SRCC and advanced SRCC, we constructed nomogram for early SRCC and advanced SRCC, respectively. Importantly, we found that LODDS had better predictive power than LNR and pN, so we constructed a nomogram based on LODDS and clinicopathological features. We also found that the predictive performance of nomogram increases over time, regardless of early or advanced SRCC. This fully shows that nomogram can effectively predict the prognosis of early SRCC and advanced SRCC, which is worthy of clinical promotion and verification.

There are still some limitations to this study. First, as a retrospective, single-center study, the results of this study still require multi-center, large-sample validation. Second, given the sample size and the fact that lymph-node metastasis is less common in early SRCC, we used the cut-off values of LODDS versus LNR proposed in previous studies, and future studies also aim to further expand the sample to explore the optimal cut-off value.

## 5. Conclusions

In conclusion, our results suggested that LODDS had a better predictive performance than LNR and pN staging regardless of early or advanced SRCC. At the same time, LODDS still has good predictive power for different numbers of retrieved lymph nodes. LODDS is an independent risk factor associated with patient prognosis, whether early or advanced SRCC. This indicates that LODDS has some application significance for SRCC.

## Figures and Tables

**Table 1:** Clinicopathologic features of the SRCC patients.

Characteristics	pT1 (*n* = 165)	pT2–pT4 (*n* = 528)	*p* Value
Sex			0.005
Male	90 (54.5)	352 (66.7)	
Female	75 (45.5)	176 (33.3)	
Age, median, range	55 (29–81)	59 (23–82)	<0.001
Tumor location			0.001
Upper	8 (4.8)	58 (11.0)	
Middle	29 (17.6)	80 (15.2)	
Lower	128 (77.6)	358 (67.8)	
Total	0 (0.0)	32 (6.1)	
Tumor size (mm)			<0.001
≤50	148 (89.7)	240 (45.5)	
>50	17 (10.3)	288 (54.5)	
pN			<0.001
pN0	130 (78.8)	86 (16.3)	
pN1	21 (12.7)	81 (15.3)	
pN2	10 (6.1)	132 (25.0)	
pN3	4 (2.4)	229 (43.4)	
pTNM			<0.001
Ⅰ	151 (91.2)	27 (5.1)	
Ⅱ	13 (7.9)	144 (27.3)	
Ⅲ	1 (0.6)	357 (67.6)	
Vascular invasion			<0.001
No	143 (86.7)	226 (42.8)	
Yes	22 (13.3)	302 (57.2)	
Neural infiltration			<0.001
No	151 (91.5)	100 (18.9)	
Yes	14 (8.5)	428 (81.1)	
LNR			<0.001
0–0.2	159 (96.4)	272 (51.5)	
0.2–0.4	5 (3.0)	128 (24.2)	
0.4–0.6	1 (0.6)	72 (13.6)	
>0.6	0 (0.0)	56 (10.6)	
LODDS			<0.001
LODDS 0	111 (62.3)	79 (15.0)	
LODDS 1	31 (18.8)	71 (13.4)	
LODDS 2	18 (10.9)	143 (27.1)	
LODDS 3	4 (2.4)	154 (29.2)	
LODDS 4	1 (0.6)	81 (15.3)	

**Table 2:** Prognostic performance of different lymph-node staging systems based on different number of retrieved lymph nodes.

	No. of LNs Retrieved			
	Total	<16	16–30	>30
	C-Index	C-Index	C-Index	C-Index
pT1 SRCC				
pN	0.676	0.635	0.965	0.674
LNR	0.728	0.635	0.958	0.674
LODDS	0.723	0.656	0.976	0.731
pT2-pT4 SRCC				
pN	0.670	0.739	0.682	0.644
LNR	0.644	0.706	0.658	0.621
LODDS	0.684	0.779	0.699	0.657

**Table 3:** Independent risk factors for prognosis for T1 SRCC.

Characteristics	Multivariate Analysis for pN		Multivariate Analysis for LNR		Multivariate Analysis for LODDS	
	HR (95% CI)	*p* Value	HR (95% CI)	*p* Value	HR (95% CI)	*p* Value
Vascular invasion		0.091		0.074		0.045
No	1		1		1	
Yes	3.254 (0.828–12.792)		3.423 (0.889–13.173)		3.789 (1.031–13.919)	
pN		0.003				
pN0	1					
pN1	0.000 (0.000-inf)	0.983				
pN2	4.067 (0.745–22.187)	0.105				
pN3	18.219 (3.950–84.030)	<0.001				
LNR				0.017		
0			1			
0.1			0.000	0.984		
>0.1			6.823 (1.831–25.427)	0.004		
LODDS						0.002
LODDS 0					1	
LODDS 1					2.207 (0.367–13.278)	0.387
LODDS 2					3.595 (0.598–21.635)	0.162
LODDS 3 + 4					21.966 (4.293–112.394)	<0.001

**Table 4:** Independent risk factors for prognosis for T2–T4 SRCC.

Characteristics	Multivariate Analysis for pN		Multivariate Analysis for LMR		Multivariate Analysis for LODDS	
	HR (95% CI)	*p* Value	HR (95% CI)	*p* Value	HR (95% CI)	*p* Value
Age	1.012 (1.011–1.023)	0.032	1.012 (1.001–1.023)	0.036	1.010 (0.999–1.021)	0.072
Tumor location		<0.001		0.002		0.006
Upper	1		1		1	
Middle	0.635 (0.405–0.996)	0.048	0.712 (0.454–1.117)	0.139	0.717 (0.457–1.125)	0.148
Lower	0.843 (0.590–1.203)	0.347	0.938 (0.655–1.343)	0.726	0.914 (0.639–1.307)	0.621
Total	1.785 (1.085–2.937)	0.023	1.878 (1.125–3.133)	0.016	1.734 (1.039–2.893)	0.035
Vascular invasion		0.381		0.189		0.314
No	1		1		1	
Yes	1.116 (0.873–1.427)		1.184 (0.920–1.524)		1.136 (0.886–1.456)	
Neural infiltration		0.026		0.023		0.023
No	1		1		1	
Yes	1.471 (1.047–2.068)		1.490 (1.056–2.102)		1.489 (1.057–2.096)	
pN		<0.001				
pN1	1					
pN2	2.917 (1.532–5.551)	0.001				
pN3	5.440 (3.012–9.825)	<0.001				
LMR				<0.001		
0–0.2			1			
0.2–0.4			2.088 (1.565–2.785)	<0.001		
0.4–0.6			2.663 (1.892–3.748)	<0.001		
>0.6			3.002 (2.056–4.385)	<0.001		
LODDS						<0.001
LODDS 0					1	
LODDS 1					2.733 (1.380–5.415)	0.004
LODDS 2					5.055 (2.751–9.289)	<0.001
LODDS 3					6.786 (3.690–12.481)	<0.001
LODDS 4					9.565 (5.040–18.155)	<0.001

## Data Availability

The data comes from the medical record system of the Cancer Hospital of Harbin Medical University. Further inquiries can be directed to the corresponding author.
